# Engagement with HIV Prevention Treatment and Care among Female Sex Workers in Zimbabwe: a Respondent Driven Sampling Survey

**DOI:** 10.1371/journal.pone.0077080

**Published:** 2013-10-15

**Authors:** Frances M. Cowan, Sibongile Mtetwa, Calum Davey, Elizabeth Fearon, Jeffrey Dirawo, Ramona Wong-Gruenwald, Theresa Ndikudze, Samson Chidiya, Clemens Benedikt, Joanna Busza, James R. Hargreaves

**Affiliations:** 1 Infection and Population Health, University College London, London, United Kingdom; 2 Zimbabwe AIDS Prevention Project Trust, Harare, Zimbabwe; 3 Centre for Sexual Health and HIV/AIDS Research (CeSHHAR) Zimbabwe, Harare, Zimbabwe; 4 Infectious Disease Epidemiology, London School of Hygiene and Tropical Medicine, London, United Kingdom; 5 Deutsche Gesellschaft für Internationale Zusammenarbeit (GmbH), Bonn, Germany; 6 United Nations Population Fund (UNFPA), Harare, Zimbabwe; 7 Population Health, London School of Hygiene and Tropical Medicine, London, United Kingdom; University of Texas Health Science Center San Antonio Texas, United States of America

## Abstract

**Objective(S):**

To determine the HIV prevalence and extent of engagement with HIV prevention and care among a representative sample of Zimbabwean sex workers working in Victoria Falls, Hwange and Mutare.

**Design:**

Respondent driven sampling (RDS) surveys conducted at each site.

**Methods:**

Sex workers were recruited using respondent driven sampling with each respondent limited to recruiting 2 peers. Participants completed an interviewer-administered questionnaire and provided a finger prick blood sample for HIV antibody testing. Statistical analysis took account of sampling method.

**Results:**

870 women were recruited from the three sites. HIV prevalence was between 50 and 70%. Around half of those confirmed HIV positive were aware of their HIV status and of those 50-70% reported being enrolled in HIV care programmes. Overall only 25-35% of those with laboratory-confirmed HIV were accessing antiretroviral therapy. Among those reporting they were HIV negative, 21-28% reported having an HIV test in the last 6 months. Of those tested HIV negative, most (65-82%) were unaware of their status. Around two-thirds of sex workers reported consistent condom use with their clients. As in other settings, sex workers reported high rates of gender based violence and police harassment.

**Conclusions:**

This survey suggests that prevalence of HIV is high among sex workers in Zimbabwe and that their engagement with prevention, treatment and care is sub-optimal. Intensifying prevention and care interventions for sex workers has the potential to markedly reduce HIV and social risks for sex workers, their clients and the general population in Zimbabwe and elsewhere in the region.

## Introduction

Female sex workers (FSWs) are a marginalised group experiencing poor health, high levels of partner abuse and other forms of violence. In some settings their HIV prevalence is very high, often 4-20 times that in the general population[1]. In sub-Saharan Africa, sex work takes many forms ranging from brothel-based activities to more informal solicitation in bars and through mobile phone and other contact networks. Despite acknowledgement of their enhanced risk, FSWs continue to be underserved by HIV programs in Africa[1]. Delivering services is complicated by the fact that sex work is illegal in countries throughout the region including in Zimbabwe[2-4]. Stigma, marginalization, and abuse of human rights have been highlighted as key determinants of reduced access to health care among FSWs in southern Africa[5]. 

FSWs were recognised in Zimbabwe's National HIV/AIDS Strategic Plan 2006-2010[6] as an important group to reach with HIV prevention and treatment services but currently there are few data to accurately estimate HIV infection rates among this group and the extent to which they are engaged with HIV prevention and care. 

In 2009, Zimbabwe's National AIDS Council (NAC) initiated a programme for FSWs[7] which provides HIV testing, syndromic management for sexually transmitted infections (STIs), contraception, primary care, health education and access to legal advice all supported by a network of peer educators. In 2011, three sites were selected for intensified programming in the form of community mobilisation of FSWs, and additional training for health care workers to reduce stigma and discrimination in health care settings. Prior to intensification of services, a survey was conducted among FSWs at these three sites to estimate their HIV prevalence and describe their engagement with HIV prevention, treatment and care services. We discuss our findings in light of potential areas to strengthen a proposed ‘treatment as prevention’ intervention for FSWs in Zimbabwe.

## Methods

### Study Population and Setting

The study was conducted in three sites in Zimbabwe; Mutare, a small city bordering Mozambique, Victoria Falls, a tourist town bordering Zambia, and Hwange, the site of a large colliery. Women were eligible for inclusion if they were aged >18 years, worked at the study site and reported that they had exchanged sex for money in the past 30 days. The surveys were conducted around 8 months after the introduction of NAC's HIV and STI prevention programme for FSWs.

### Sampling

Rapid ethnographic mapping to determine the spatial and social organisation of sex work was conducted at each site to identify key geographic areas where sex work was being conducted, the main typologies of sex work, and inform preliminary estimates of the size of the FSW population. 

A bio-behavioural survey was conducted at each site. Respondents were recruited to the survey through respondent-driven sampling (RDS). This variant of chain-sampling can be used to recruit research participants from “hidden” populations. In contrast with other chain sampling approaches, RDS rations the number of recruits per respondent, tends to deploy a larger number of waves of recruitment, and provides financial incentives to recruiters[8]. The aim is to recruit a representative sample of research participants of the hidden population of which the respondents are members. There is ongoing debate in the literature about the extent to which RDS achieves this aim and the conditions under which this may be more or less likely[9-11]. Nevertheless, RDS offers a systematic and theoretically grounded approach to recruiting hidden populations.

The initial recruiters (seeds) were FSWs with close links with other FSWs. The seeds were purposively selected so as to represent a range of ages, geographic areas and sex-work typologies identified during preliminary mapping. Ten seeds were recruited from Mutare and six each from Hwange and Victoria Falls. 

Each seed respondent completed a questionnaire, had a finger prick blood sample collected for HIV antibody testing, and was issued with two uniquely identified coupons. The seeds passed these coupons on to individuals meeting the study inclusion criteria, and on presenting a coupon to study staff these individuals were asked to give consent and be recruited to the study. These recruits were themselves given two coupons to refer further peers, and so on. Each respondent was provided with US$5 to cover costs of participation. A further US$2 was provided to the recruiter for each successfully recruited referral. Six waves of coupons were issued. In Mutare, up to 630 individuals could be recruited if all coupons resulted in referral and 378 each in Hwange and Victoria Falls. These “total possible” numbers were roughly double initial estimates of the size of the sex work population at each site. We chose to limit each respondent to two referral coupons to increase the number of waves since we anticipated that estimates would stabilise after 4-5 of our potential 6 waves of recruitment.

Voluntary counselling and testing was made freely available for those participants who wanted to know their HIV results.

### Data collection

The questionnaire was developed in English and translated into Shona and Ndebele, the local languages. It was piloted before being implemented to check that all wording was understood and unambiguous. Responses were compiled directly into a computer-assisted survey instrument (QDS™ Nova research Company) by trained female interviewers. The questionnaire data were collected anonymously and included information on socio-demographic and economic characteristics, sexual behaviour, psychological health, physical health, past history of STIs, sexual and social networks, social capital, utilisation of services including HIV testing, provision of antiretroviral therapy (ART), prevention of mother to child transmission (PMTCT) and contraception. Where appropriate we used questions that had been previously used or validated in Zimbabwe or elsewhere[12-15]. The questionnaire took an average of 40 minutes to complete.

### Laboratory procedures

Finger prick blood samples were collected[16] by nurse counselors and transported to the National Microbiology Reference Laboratory in Harare. They were tested for HIV-1 antibody in series using AniLabsytems EIA kit (AniLabsystems Ltd, FIN-01720, Vantaa, Finland) with those specimens testing positive also tested using Vironostika® HIV Microelisa System bioMerieux, Inc., Durham NC 27704. Discrepant results were resolved using western blot. 

### Key variables

We identified a set of primary outcome variables: laboratory-confirmed HIV status, reported HIV testing within the past 6 months among those HIV negative, and whether currently taking ART among those HIV positive. We explored discrepancies in self-reported and laboratory-confirmed HIV status.

We also investigated consistent (“always”) condom use with commercial clients and with steady/permanent non-commercial partners. The question used to assess condom use was “In the past month, how often did you use condoms with your steady partner?” Participant responses were grouped as “Always” and “Not always”. 

The variable used to estimate network degree was "how many of those sex workers whom you know personally would you consider recruiting into this study?” 

### Statistical analysis

We first used unadjusted statistics to describe the population. All of our analyses were stratified by site. As recommended for RDS, we excluded seeds from the analysis. 

Our analysis sought to account for the RDS design of the study, while recognising that there remains debate about which methods best achieve this. It is theorised that successive waves of recruits should become more representative of the source population. Individuals may recruit individuals more like themselves than would be expected on average. To explore these issues we assessed whether the prevalence of key outcome measures stabilised over waves of recruitment and whether socio-demographic characteristics of recruiters were associated with characteristics of their recruits. 

To estimate outcome prevalences we first used the *Stata* RDS package [17] The multiplicity estimate of average network degree was used to correct for over-representation of those with large degree in the sample. Bootstrapping with 1000 repetitions was used to obtain percentages and standard errors except for subgroup analyses, for which Taylor linearization was used. Next, we adapted a method proposed by Szwarcwald to estimate predicted prevalences of the key outcome variables in the source population[18]. We used random effects logistic regression for this purpose. We fitted models including weighting for the inverse of participant degree (using the same question as for RDS analysis). We also specified a random effect term for “seed” such that the variance of responses from individuals who were part of the same recruitment chain was modeled to take some account of potential clustering. 

### Institutional Review Board approval

All participants gave written consent collected according to the principles of Good Clinical Practice. Institutional Review Board approval (IRB) for the study was given by the Medical Research Council of Zimbabwe (MRCZ) The UCL Ethics Committee. The consent form was approved by all IRBs in English and the translations to Shona and Nedebele were approved by MRCZ.

## Results

### Recruitment

**Figure 1:**
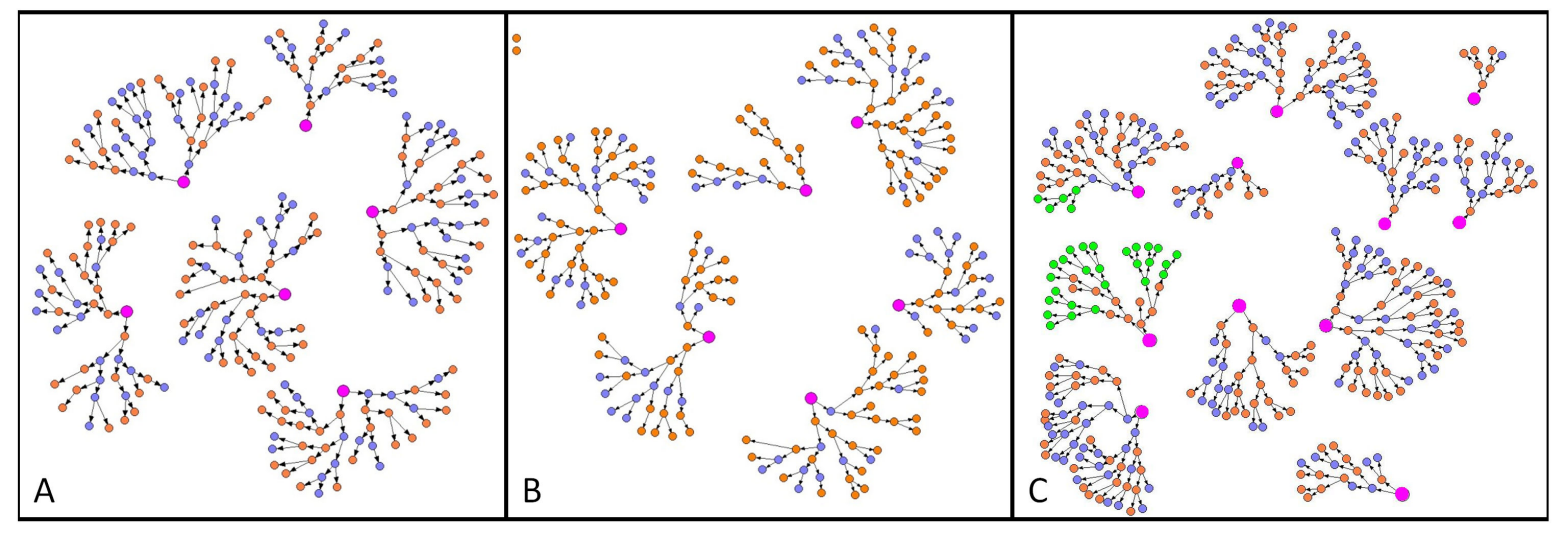
Chains of recruitment in the RDS sites for Hwange (A), Victoria Falls (B) and Mutare (C). Each circle represents a participant in the survey and the lines represent the passing of recruitment coupons. Orange circles represent women who tested HIV positive and blue circles HIV negative. The green circles in Mutare represent women for whom the HIV status is missing. Finally, the pink circles represent the seeds.

Over six rounds of recruitment 370 FSWs were recruited in Mutare, 237 in Hwange and 229 in Victoria Falls. All 22 seeds recruited women into the study. The recruitment chains are depicted in Figure 1. One batch of dried blood samples from 32 individuals was misplaced by the laboratory and so were not tested for HIV, these individuals were associated with two recruitment chains in Mutare (also shown). 

**Figure 2:**
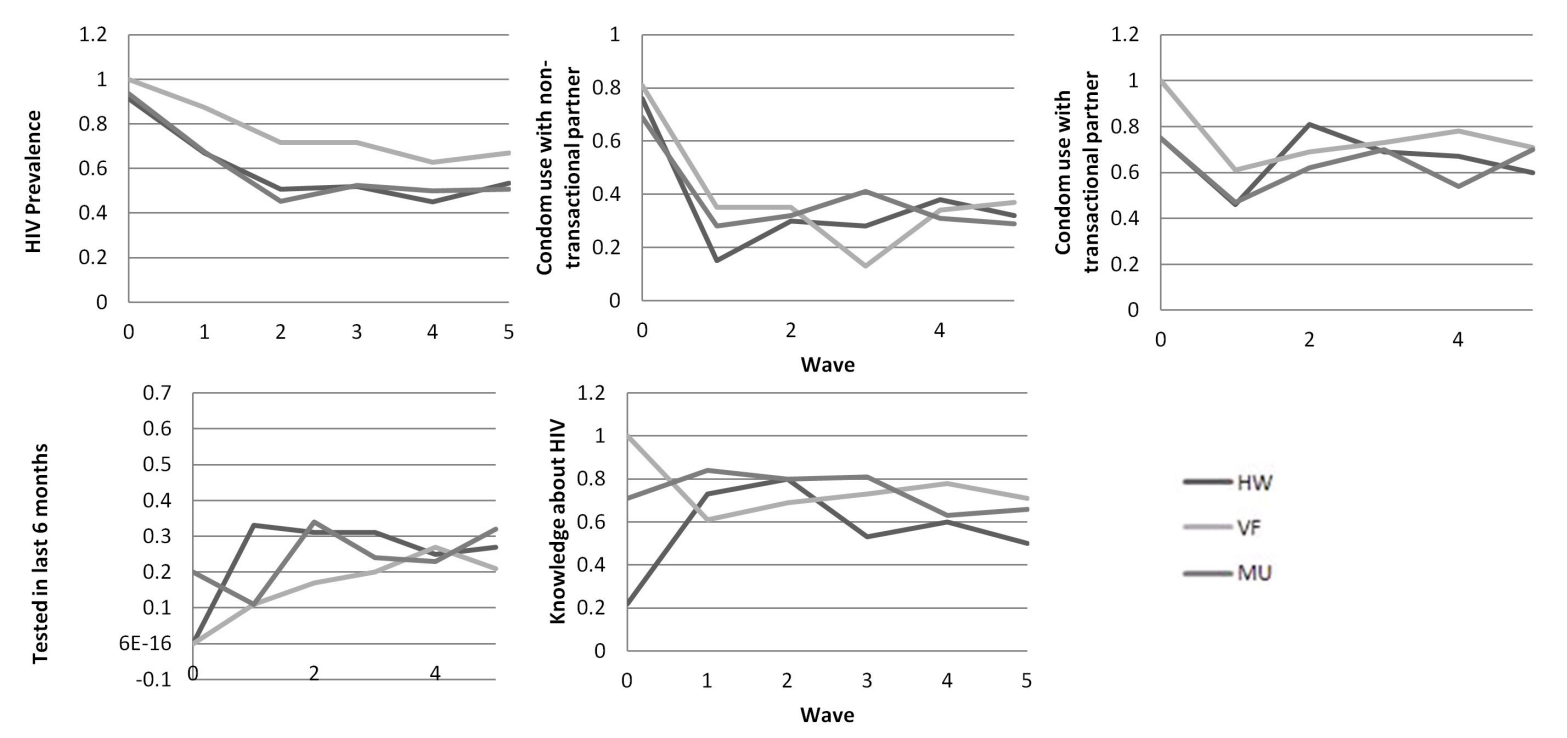
RDS validation: Outcome measure estimates over waves of recruitment.

About 90% of seeds in Mutare and Hwange and all the Victoria Falls seeds were HIV positive (Figure 2). HIV prevalence was higher in the first wave of recruitment before falling to a roughly stable prevalence in subsequent waves. Seeds were more likely than recruits in all waves to report consistent use of condoms in all three sites, but there was no discernible pattern in consistent condom use by wave after the first wave of recruits. There was no consistent trend in HIV testing in the previous 6 months by wave in the three sites. Figure 2 suggests that the number of waves required to reach equilibrium was less than the number of waves in the study for all of the outcomes. Later waves also include more women. There was little evidence for strong association between socio-demographic characteristics of recruiters and outcomes in their recruits. The associations for which there was some evidence of autocorrelation were different across sites. There were no associations in any site that showed strong evidence for autocorrelation between recruiters and recruitees (p values of less than p=0.01). 

### Characteristics of sampled population

In the three sites FSWs were on average 27-30 years old, and in Mutare and Hwange had generally been long term residents (Table 1). The majority in all sites were widowed, or divorced from a partner (68.3%-75.0%). FSWs in Hwange and Mutare had longer duration in sex work than those in Victoria Falls. Most solicited clients in bars, with significant numbers also soliciting by phone or at home or on the street. In all sites, 17 years was the median age of first sex, and 22-23 years the median age of first commercial sex. Client load was highest in Victoria Falls (median 14 in the last month) and lowest in Hwange (6). FSWs reported a median of 2 commercial partners in the past week, most of whom were new clients. A minority reported anal sex, though among those, 35-58% reported it in the last month. Most participants had at least one close friend who was a sex worker, with 2-4 sex worker close friends most common in all three sites (62.4% of participants in Mutare, 44.5% in Hwange and 58.0% in Victoria Falls). 

**Table 1:** Sociodemographic characteristics of sex workers in three sites in Zimbabwe, RDS weighted.

		Mutare (n=370)		Hwange (n=237)		Victoria Falls (n=229)	
		n	% (95% CI)	n	% (95 % CI)	n	% (95 % CI)
**Age**	<25 years	97	30.0 (22.8-37.2)	57	28.4 (19.2-37.6)	80	39.5 (30.3-48.7)
	>25≤30	75	19.0 (13.5-24.5)	50	21.5 (14.8-28.2)	62	24.9 (16.9-32.9)
	>30≤35	74	20.1 (14.8-25.4)	44	19.2 (12.4-26.0)	45	15.9 (9.5-22.4)
	>35≤40	46	12.3 (7.5-17.1)	41	15 (9.7-20.3)	19	7.3 (3.4-11.2)
	>40	78	18.0 (12.6-23.4)	45	15.9 (9.9-21.9)	23	12.3 (6.2-18.4)
**Marital**	Single, never married	64	23.4 (16.9-29.8)	77	24.5 (19.0-30.2)	68	29.5 (21.8-37.3)
**Status**	Married or Living together	9	3.4 (0.9-6.6)	1	0.5 (0.1-1.3)	2	2.2 (0.6-5.7)
	Divorced	126	56.1 (47.9-64.4)	216	57.4 (51.1-64.2)	118	46.0 (38.3-54.4)
	Widowed	38	17.0 (10.7-24.2)	75	17.6 (12.1-23.5)	41	22.3 (13.5-31.2)
	Don't know	0	0 (0-0)	1	*	0	0 (0-0)
**Education**	None or incomplete primary	49	12.9 (8.4-17.4)	21	12.7 (7.1-18.3)	34	15.5 (9.1-21.9)
	Incomplete secondary	186	49.7 (42.8-56.6)	118	55.3 (46.9-63.8)	146	67.5 (59.1-75.9)
	Complete secondary or higher	134	37.3 (30.4-44.2)	87	32.0 (24.1-39.9)	49	17.0 (10.3-23.7)
**Duration of sex work**	<2 years	42	9.8 (5.6-14.1)	38	18.3 (12.0-24.6)	59	31.2 (22.8-39.6)
	>2<4 years	102	33.5 (25.9-41.1)	70	29.3 (21.0-37.6)	76	30.5 (22.1-38.9)
	>4<6 years	59	14.3 (9.7-18.9)	37	16.9 (9.6-24.1)	39	13.4 (7.3-19.5)
	>6<10 years	70	18.4 (13.4-23.4)	35	13.8 (7.8-19.8)	32	17.1 (10.6-23.6)
	>10 years	97	24.1 (18.5-29.7)	57	21.8 (14.8-28.8)	23	7.9 (3.6-12.2)
**Duration at that site**	<5 years	60	20.7 (14.3-27.1)	57	25.04 (17.5-32.6)	74	40.1 (30.5-49.7)
	>5<10	53	14 (9.4-18.6)	28	16.01 (8.7-23.3)	52	21.8 (14.1-29.6)
	>10<20	66	14.7 (9.8-19.6)	32	14.9 (7.7-22.2)	57	20.0 (13.0-27.0)
	>20<40	153	43.2 (35.7-50.8)	97	36.3 (27.8-44.7)	44	18.1 (11.7-24.4)
	40+	38	7.3 (3.8-10.8)	23	7.7 (3.3-12.2)	2	**
**Where do they solicit clients**	Bars/nightclubs/entertainment	298	80.7 (75.5-85.9)	192	80.5 (74.2-86.8)	217	94.5 (90.7-98.3)
	Telephone/at home	106	26.8 (20.7-32.9)	89	38.9 (30.9-46.9)	42	15.8 (9.0-22.6)
	Market Stalls	32	8.2 (4.8-11.6)	6	3.5 (0.4-6.6)	2	0.4 (-0.2-1.0)
	On the street	133	35.5 (29.4-41.6)	71	26.0 (18.7-33.3)	30	9.8 (5.7-13.9)
	Lodges	30	5.9 (3.1-8.7)	18	5.0 (2.4-7.7)	12	2.0 (0.4-3.6)
	Hotels	19	6.2 (3.1-9.3)	11	2.4 (0.7-4.2)	14	3.4 (1.0-5.8)
	Other	32	7.5 (4.4-10.6)	20	7.3 (3.8-10.9)	6	1.7 (-1.2-4.6)
**No. of commercial partners in last week**	0	30	7.5 (4.5-11.0)	25	12.9 (6.7-17.8)	19	7.7 (3.3-12.8)
	1-3	146	45.3 (38.3-52.0)	142	64.7 (57.2-72.0)	98	41.8 (32.6-50.9)
	4-9	140	36.4 (30.1-42.9)	54	18.6 (13.0-24.7)	82	39.2 (30.8-48.7)
	10+	54	10.8 (7.6-14.5)	16	4.6 (2.3-7.7)	30	11.3 (6.1-17.1)
**How many times stopped by police in last week**	0	154	39.3 (32.1-46.7)	150	66.5 (58.9-73.8)	70	33.5 (25.7-41.6)
	1-4	164	49.4 (42.1-56.7)	28	27.0 (20.1-34.5)	92	41.3 (33.4-49.5)
	5+	52	11.4 (7.6-15.7)	19	6.6 (3.4-9.8)	67	25.2 (18.8-32.7)
**No. of FSWs who are close friends**	0	8	2.5 (1.0-4.2)	22	9.1 (4.8-14.2)	11	4.6 (16.7-7.7)
	1	56	19.6 (13.3-26.4)	76	34.5 (27.1-42.5)	60	28.8 (19.6-37.4)
	2-4	214	62.4 (55.4-69.3)	108	44.5 (36.6-53.0)	133	58.0 (49.0-68.1)
	5+	92	15.6 (11.5-20.0)	31	11.9 (6.9-18.1)	25	8.7 (4.7-13.9)

* Included 'don't know' with married in order to calculate weighted estimates (too few observations to estimate weighting otherwise).

** Combined with >20<40 category as too few observations in the response category to calculate weighted estimates.

Most FSWs (72-77%) reported having a “permanent partner” with whom they had had sex 4.5-6 times in the last month (A steady or permanent partner was defined as a husband, or a boyfriend with whom there is an expectation of commitment to a longer term relationship).

Levels of intimate partner violence (IPV) were high with 13-24% reporting physical and 8-23% reporting sexual violence in the last year. IPV was more common within permanent partnerships than with clients. Many FSWs reported they had experienced harassment or abuse from the police. The percentage of FSWs who had been stopped by the police at least once in the previous month was lower in Hwange at 33.6% than in Mutare (60.7%) or Victoria Falls (66.5%). 27-32 percentage points of FSWs reported recent experience of genital sores, ulcers or unusual genital discharge, with about 66% having sought treatment for this. 

### Outcome variables

The approaches to calculating prevalence estimates for the outcome variables made a small difference to the estimates obtained. For example, in Mutare and Hwange, the weighted HIV prevalence was slightly lower than the unweighted, while in Victoria Falls weighted results were slightly higher. The RDS weighted prevalence and the regression weighted prevalence were similar for each of the outcomes except for the percentage testing for HIV in the previous 6 months (table 2). 

**Table 2:** Main outcome measures.

**Mutare**	n / N	Unweighted %	RDS weighted % and 95% confidence intervals	Regression predicted weighted % and 95% confidence intervals
HIV Prevalence	188/338	55.6	50.6 (43.5-58.6)	52 (45.1-58.8)
Consistent condom use - transactional partners	242/361	67	65.7 (58.4-71.2)	65.2 (58.3-71.5)
Consistent condom use - non transactional partners	96/271	35.4	33.3 (26.5-40.9)	33.6 (30-37.4)
HIV testing within last 6 months*	73/262	27.9	30.3 (23.2-38.6)	30.2 (22.2-39.6)
**Victoria Falls**				
HIV Prevalence	156/229	68.1	69.6 (61.7-76.7)	70 (61.0-77.7)
Consistent condom use - transactional partners	160/220	72.7	71.6 (63.2-78.7)	72.6 (62.8-80.5)
Consistent condom use - non transactional partners	60/166	36.1	31.3 (23.1-40.9)	33.3 (22.4-46.4)
HIV testing within last 6 months (HIV negatives only)	31/146	21.2	13.4 (8.7-19.9)	13.2 (10.8-16.1)
**Hwange**				
HIV Prevalence	136/237	57.4	50.6 (41.7-59.0)	51.8 (46.5-56.9)
Consistent condom use - transactional partners	140/220	63.6	65.7 (57.3-73.3)	66.3 (57.6-74.1)
Consistent condom use - non transactional partners	62/185	33.9	33.4 (24.8-42.6)	34.2 (24.2-45.8)
HIV testing within last 6 months*	46/167	27.5	25.1 (17.4-34.7)	25.6 (17.9-35.2)

* Amongst only those who did not report previously testing HIV positive

The majority of FSWs recruited were HIV positive. HIV prevalence was highest among FSWs recruited in Victoria Falls with RDS-weighted prevalence of 69.6% and a regression weighted prevalence of 70.0%. In Mutare, 50.6% (RDS) - 52% (regression) of FSWs were HIV positive, while in Hwange, this figure was similar at 50.6% (RDS) - 51.8% (regression). 

**Figure 3:**
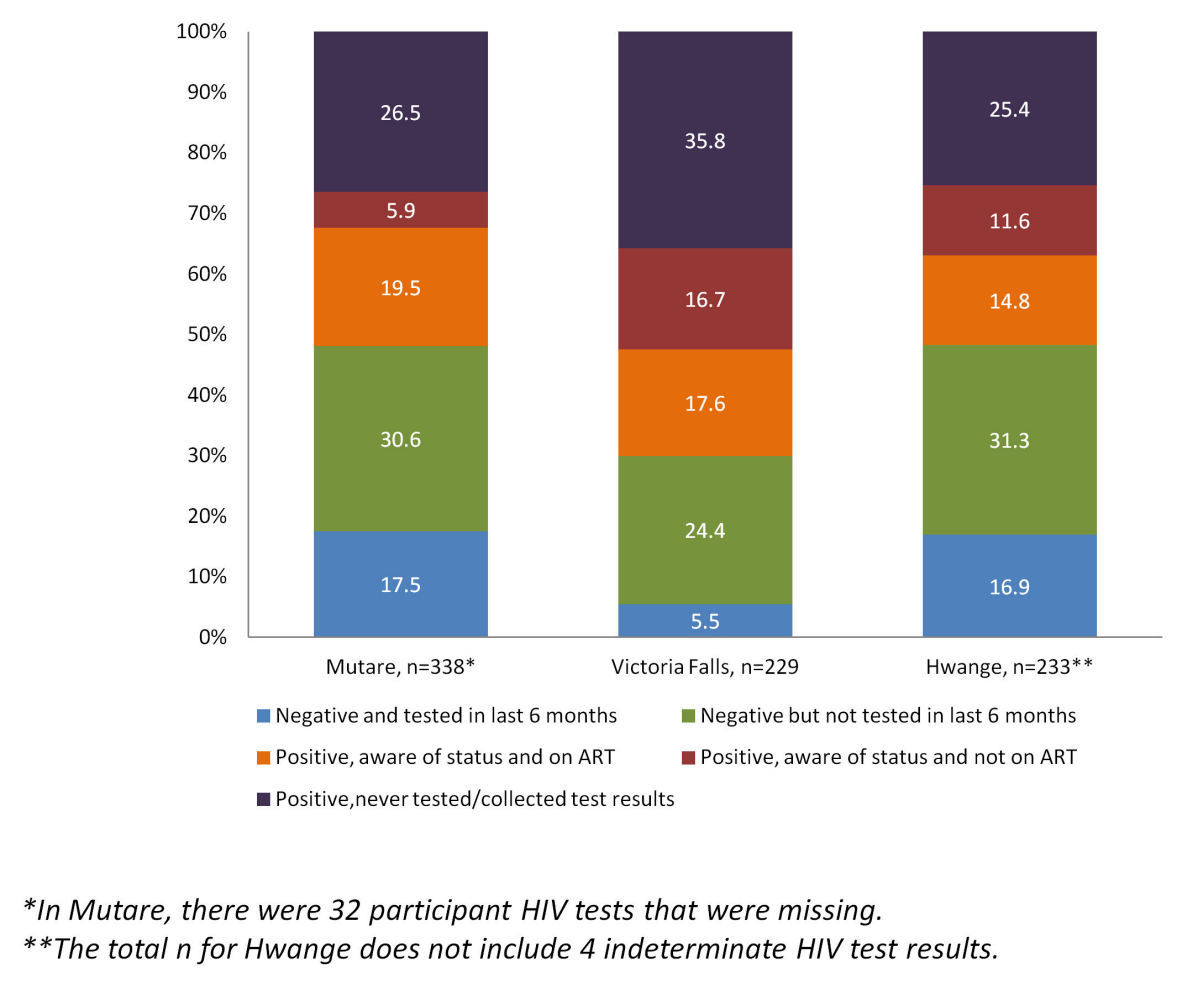
Previous knowledge of HIV status.

**Figure 4:**
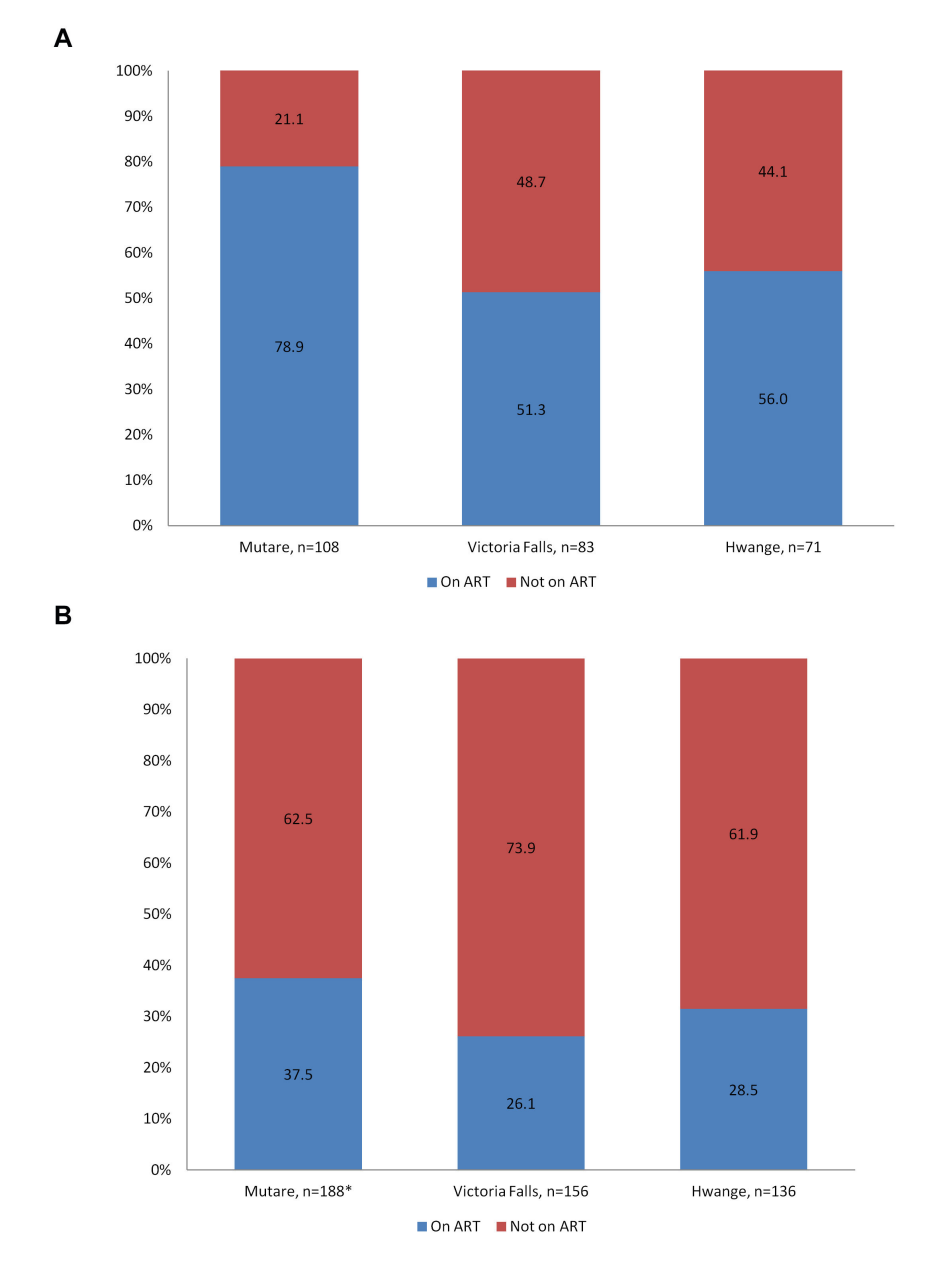
Percentage of HIV positive female sex workers who knew their status and were on ART (a). Percentage of *all* HIV+ female sex workers who reported that they were on ART (b).

Because we had information on testing, self-reported HIV status amongst FSWs and laboratory confirmed serostatus in the whole sample, we were able to investigate the proportion of FSWs who were aware of their true HIV status and who were aware and on treatment (Figure 3). The category of most concern is FSWs with laboratory-confirmed infection but who are unaware of their status, either because they had never tested, their status had since changed or because they never collected the results of their test. In all three sites, this category was approximately half of all HIV positive participants (percentages are weighted by degree), see Figure 3. Of those who reported in the questionnaire that they were HIV positive, between 51% and 74% reported being on ART (see Figure 4a). This means that of all those with laboratory-confirmed HIV infection, the majority (61.9% to 73.9%) were not on ART (see Figure 4b). Of those who were HIV negative, the majority (65-82%) were also unaware of their status (see Figure 3). This was either because they had not tested in the last 6 months, because they never collected their results or because, in the case of Mutare only, 8 had previously thought they were HIV positive. Only 21-28% of self-reported HIV negative individuals reported having had an HIV test in the last 6 months.

The most common location of last HIV test amongst those who had ever tested was the hospital (38.7% in Mutare, 58.8% in Victoria Falls and 63.2% in Hwange). In Hwange and Victoria Falls, almost all those who reported that they were on ART were receiving treatment at the hospital (42/43 and 41/44 respectively). In Mutare, 55.5% were receiving treatment through the primary care ART clinic while 41.2% were treated at the hospital. 

In all sites, FSWs were more likely to report that they always used condoms with commercial partners than with steady/permanent partners. The regression-weighted prevalences of consistent condom use with transactional partners were 65.2% (Mutare), 72.6% (Victoria Falls) and 66.3% (Hwange). For consistent condom use with steady/permanent partners, prevalence was 33.6%, 33.3% and 34.2% respectively. 

## Discussion

This is one of the first systematic surveys of HIV and associated factors among FSWs to be conducted in Zimbabwe. Of note we explored rates of engagement in HIV prevention, treatment and care, critical if UNAIDS goal of zero new infections and zero deaths are to be achieved[19]. We recruited FSWs from a tourist town, a mining town and Zimbabwe's third city. The FSWs we recruited were at extremely high risk of HIV with a prevalence of HIV between 50-70%, 3-4 times that of the women in the general population. While the majority of those who reported that they were HIV positive on the questionnaire said they were accessing ART services, overall only 26-38% of those with laboratory-confirmed infection (60% of HIV positives in Zimbabwe are estimated to be eligible for ART using current National Guidelines). Few of those who were positive but were unaware of their diagnosis reported having tested recently. Although reported condom use was high the majority of women reported that they had not used condoms at each recent sexual encounter either with client or regular partners. 

This study confirms that FSWs in Zimbabwe have a much higher risk of HIV than those in the general population[1] in addition they report high rates of symptomatic STIs likely to further increase risk of contracting and transmitting HIV[20]. HIV prevalence rates as high as 60-90% have been reported for sex workers in Kenya[21], Central and East Africa[22], and Rwanda[23]. Importantly, over the past 10 years, Zimbabwe has experienced a well-documented decline in HIV prevalence in the general population with the prevalence in women now around 15%[15,24,25]. By contrast the prevalence among these FSWs remains at the very high rates found much earlier in the epidemic[26] suggesting that they remain relatively untouched by prevention services.

The majority of women recruited to this study were unaware of their HIV status. Historically data on sex worker rates of HIV testing are scant, although sub-optimal when reported, e.g. 4% of FSWs surveyed in Somalia in 2008 had ever tested[27], and 38% in the Democratic Republic of Congo in 2005-6[28]. Previous studies have found that barriers to testing include lack of awareness of services, distance to facilities, transportation costs, opportunity costs, time constraints, and fear of a positive result[29-31]. Barriers unique to sex work include anxiety about contact with authorities and concern about confidentiality, particularly that other FSWs or potential clients may learn their status[32]. 

Learning one’s HIV status is a prerequisite for entry into HIV care, and there is evidence that people modify their behaviour to engage in less high risk behaviour after testing HIV positive[33-37]. There have been successful interventions to increase HIV counselling and testing among FSWs[38]. Furthermore, strengthened peer support and a more cohesive social environment are associated with sex workers’ willingness to engage in care (testing, treatment initiation and adherence)[39,40]. WHO recommends that retesting ‘should be at least annually’ for those at high risk[41]. There is little evidence on FSWs’ uptake of and retention in HIV treatment programs in Africa; little is known about their progression through the “care cascade” compared to HIV positive clients in the general population.

Of those FSWs who reported that they were HIV positive in the questionnaire, Figure 3 shows that the majority also reported that they were on anti-retroviral therapy (ART). From a public health perspective, it is important to also know what percentage of all HIV positive sex workers, including those unaware of their status, were on ART. Figure 3 shows that for Mutare, this figure was 37.5%, for Victoria Falls it was 26.1% and for Hwange, 28.5%. 

Although it is well documented that FSWs face numerous barriers to health seeking, relatively little has been written about their ability to engage with HIV prevention and care services. These data suggest that while reported engagement is relatively high among those who are accurately aware of their status, the majority are not accessing prevention and care services. We have shown previously that Zimbabwean FSWs attending general health services experience high levels of stigma and discrimination [42]. Programmes to increase FSWs engagement with services are critical both for their own wellbeing and likely for the wider public health. There is some evidence that community mobilisation, which seeks to empower women individually and collectively, can increase condom usage and improve uptake of health services and it is now recommended by WHO[43]. What is less clear is whether it is cost effective to include treatment and care for HIV positive FSWs within sex worker specific services (and pre-exposure prophylaxis to HIV negative women). Mathematical modeling suggests that there are potential benefits of intensifying treatment among FSWs in generalized epidemics[44] but empirical data to support this is lacking.

This survey has some limitations. The recruitment of survey participants through RDS poses several challenges to analysis and there remains debate about the methods of analysis. As in all applications of RDS it was not possible for us to empirically verify the extent to which the sample we recruited reflects the characteristics of FSWs working in the three sites. Nevertheless, we deployed several approaches to estimating the prevalence of key outcome variables and while there were some differences in our estimates these were minor. The characteristics of participants appeared to converge over waves of recruitment as would be expected if later waves are more likely to approximate the source population. We found a random effects regression approach to be the most flexible with regard to missing data and providing an approach to dealing with potential homophily, though evidence for homophily was weak. We had high rates of acceptance to participate in the survey. 

The survey would have been strengthened if we had been able to collect samples for CD4 count testing among those infected with HIV (to determine what proportion of those eligible for care were accessing it). In addition, it would have been helpful to determine the proportion of women with STI diagnoses as STI symptoms correlate poorly with diagnosis in women[45]. The proportion of FSWs that were recruited early in their sex work careers was relatively low in Mutare and Hwange.

This survey suggests that prevalence of HIV is exceedingly high among FSWs in Zimbabwe and that their engagement with prevention, treatment and care is sub-optimal. In addition, women suffer high rates of intimate partner violence, police harassment and discrimination by the wider community. Intensifying interventions is likely to markedly reduce HIV and social risks for FSWs, their clients and the general population in sub-Saharan Africa [46]. We plan to assess the impact of the increasing scope of Zimbabwe's National Sex Work on HIV and other social outcomes for both FSWs and the population more widely. 

## Supporting Information

Table S1 **Respondent driven sampling questionnaire – English version.**
(XLSX)
